# NGAL in the Development of Acute Kidney Injury in a Murine Model of Remote Ischaemic Preconditioning and Liver Ischaemia Reperfusion

**DOI:** 10.3390/ijms25105061

**Published:** 2024-05-07

**Authors:** Esther Platt, Francis Robertson, Ali Al-Rashed, Riko Klootwijk, Andrew Hall, Alberto Quaglia, Alan Salama, Lauren Heptinstall, Brian Davidson

**Affiliations:** 1 Division of Surgery and Interventional Science, University College London, London NW3 2PF, UK; esther.platt.20@ucl.ac.uk (E.P.); francis.robertson.13@ucl.ac.uk (F.R.); 2 Department of Renal Medicine, University College London, London NW3 2PF, UK; ali.al-rashed.19@ucl.ac.uk (A.A.-R.); a.salama@ucl.ac.uk (A.S.); 3 Department of Cellular Pathology, Royal Free London NHS Foundation Trust, London NW3 2QG, UK

**Keywords:** ischaemia reperfusion, acute kidney injury, remote ischaemic preconditioning

## Abstract

Acute kidney injury (AKI) is common following liver transplantation and is associated with liver ischeamia reperfusion (IR) injury. The purpose of this study was to use a mouse model of liver IR injury and AKI to study the role of Neutrophil Gelatinase Associated Lipocalin (NGAL), a biomarker of AKI, in liver IR injury and AKI. We demonstrate an adapted, reproducible model of liver IR injury and AKI in which remote ischemic preconditioning (RIPC) by repeated episodes of hindleg ischemia prior to liver IR reduced the severity of the IR injury. In this model, serum NGAL at 2 h post reperfusion correlated with AKI development early following IR injury. This early rise in serum NGAL was associated with hepatic but not renal upregulation of NGAL mRNA, suggesting NGAL production in the liver but not the kidney in the early phase post liver IR injury.

## 1. Introduction

Ischaemia reperfusion (IR) injury, the injury that occurs when the blood supply to an organ is interrupted and reconstituted, is a key mechanism of organ damage in solid organ transplantation [1]. IR injury occurs in 2 phases. In the first phase, ischaemia leads to depletion of energy reserves within cells and the build-up of toxic metabolites, including free radicals [2]. In the second phase, reperfusion restores the oxygen supply to the ischaemic tissue and is accompanied by dysregulation of cellular processes, mitochondrial injury, cell fragmentation, and initiation of cellular death mechanisms [3]. The influx of immune cells and upregulation of the systemic response potentiates the organ injury [4].

IR injury is a key determinant in the viability of organs and recipient survival post organ transplantation [5,6,7]. The transplantation research has long been centred on the need to either obviate injury or attenuate the recipient’s systemic response to injury in a transplanted organ [8,9,10,11]. One potential strategy for the attenuation of a recipient’s response is Remote Ischaemic Preconditioning (RIPC). In RIPC, either the donor or the recipient is exposed to a reduced and sometimes repeated ischaemic stress of a anatomically distant tissue, with a phase of reperfusion between stresses and prior to retrieval or implantation of the transplant organ [12]. Evidence from a variety of different experimental and clinical settings suggests that this approach can reduce the systemic inflammatory response and downregulate IR injury [13,14,15,16,17,18]. The underlying mechanisms of protection remain poorly understood but involve neural, humoral and systemic pathways and include a downregulation of oxidative stress and upregulation of protective pathways within the transplant organ [19]. Whilst experimental animal models have provided promising results, there has been limited translation into clinical practice [20,21,22,23,24].

The kidneys are known to be especially vulnerable to injury following heart or liver transplant [25,26]. Acute kidney injury (AKI) is seen in approximately 50% of patients who undergo orthotopic liver transplantation (OLT) [27]. The degree of liver IR injury following OLT correlates with the severity of post-operative AKI and a requirement for renal replacement therapy (RRT) [28]. AKI post OLT is associated with graft failure, worse overall survival, delay to discharge from both intensive care and hospital, and progression to chronic kidney disease, including end-stage renal failure (ESRF) [27,29,30].

Neutrophil Gelatinase Associated Lipocalin (NGAL) is increasingly used as an early biomarker of renal injury in animal and clinical studies of AKI [31,32]. NGAL is an iron binding protein that was first isolated from secondary granules in neutrophils and may exist as a monomer, dimer, or heterodimer (with MMP-8) [33]. Its principle physiological role is believed to be sequestration of free iron, reducing the availability of iron as an energy source to bacteria, as part of the innate defence to infection [34]. NGAL has since been isolated from a variety of other cell and tissue types and is believed to have additional functions, but these have not been fully elucidated [35,36]. NGAL is usually detected in clinical samples using ELISA tests on either plasma, serum, or urine samples. In the experimental setting, investigators have used a combination of ELISA, Western Blot, and mRNA quantification to evaluate changes in NGAL expression in response to a variety of stimuli.

We previously showed that the urinary NGAL levels measured at the end of liver transplant surgery accurately predict both development of AKI and renal replacement therapy (RRT) requirement post OLT [37]. The origin of NGAL in this context is not clear, and there is no clear consensus in the literature about the origin of NGAL in other forms of AKI, with some authors suggesting a renal origin and others an immune cell origin [38,39]. It is also unclear whether NGAL is released as part of the response to IR injury or whether it has a pathological role.

The purpose of this study was to adapt an established murine model in which liver IR injury is known to cause AKI to study the role of NGAL in the early phase post liver IR injury.

## 2. Results

### 2.1. The Mouse Model of Liver Ischaemia Reperfusion Was Associated with Liver and Renal Injury

**Figure 1:**
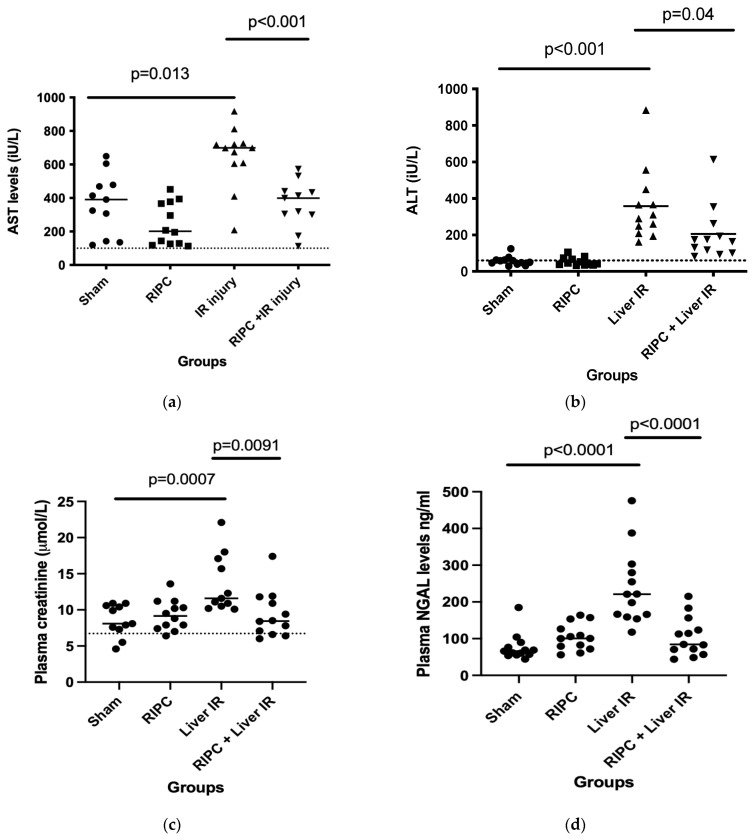

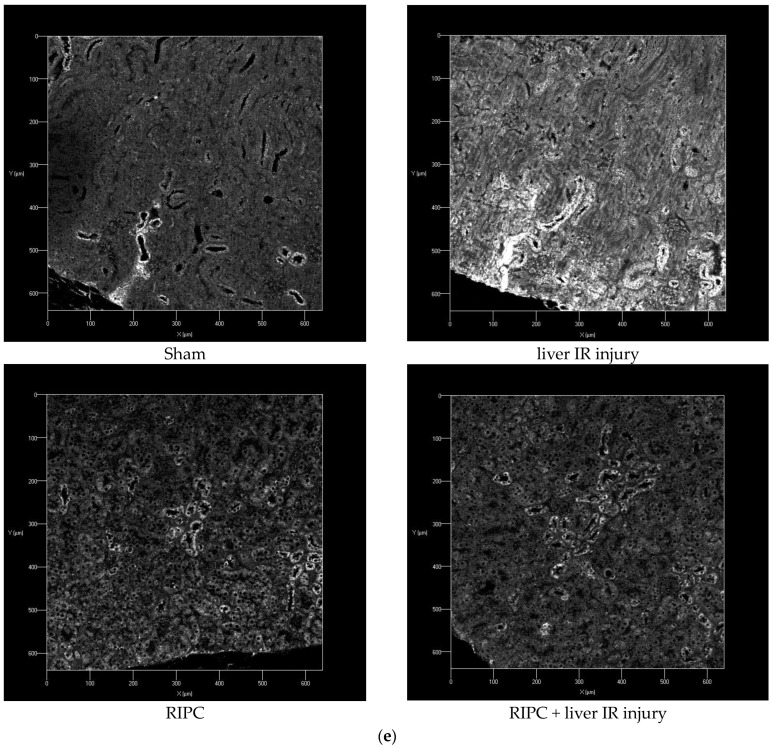
(**a**–**d**) Biochemical markers of liver and renal injury at 2 h post liver reperfusion. Plasma transaminases (AST and ALT) measured at two hours post liver reperfusion were used as surrogate markers of liver injury and confirmed injury in the liver Ischaemia Reperfusion (IR) group (**a**,**b**). Liver IR was provided by a single episode of 45 min of clamping the left and middle portal pedicles followed by 120 min of reperfusion. Liver injury was attenuated by pre-treatment with Remote Ischaemic Pre-Conditioning (RIPC) in the form of intermittent clamping of the femoral vessels for 5 min episodes with reperfusion in between and was not seen in either the sham laparotomy or RIPC only groups. Liver IR injury was accompanied by renal injury, demonstrated by a statistically significant increase in plasma creatinine compared to the sham laparotomy ((**c**), *p* = 0.0007). Renal injury was attenuated by pre-treatment with RIPC. Plasma Neutrophil Gelatinase Associated Lipocalin (NGAL) concentration at two hours post reperfusion was significantly elevated in the Liver IR group but was reduced to control levels with RIPC pre-treatment (**d**). Dotted lines denote the normal values for plasma AST, ALT, and creatinine in mice. (**e**) Histological evidence of liver injury. Histological specimens confirmed oxidative injury with increased staining for DNA/RNA oxidative damage in renal specimens from the liver IR group compared to the sham; injury was attenuated by RIPC (**e**). *N* = 12 mice/experimental group for biochemical measurements.

The murine model of partial liver ischaemia (provided by 45 min of continuous clamping of the left and middle portal pedicles followed by 120 min of reperfusion) was associated with the development of ischaemia reperfusion injury, as indicated by the elevated plasma AST/ALT levels at two hours post reperfusion (Figure 1). Remote ischaemic pre-conditioning (RIPC) alone was not associated with liver injury (RIPC provided by intermittent 5 min episodes of clamping the right femoral pedicle with reperfusion in between). RIPC prior to liver ischaemia reperfusion was associated with a significant reduction in liver injury (reduction in AST *p* ≤ 0.001, ALT *p* = 0.04). Morphological evidence of injury was not demonstrated at this early time point on standard H&E histological staining (Appendix A). Ischaemia reperfusion of the liver produced acute kidney injury (Figure 1c), as measured by serum creatinine at two hours post reperfusion. There was no morphological evidence of renal injury in the standard H&E histological staining results at this early time point (Appendix A) but staining with a DNA/RNA damage antibody (15A3) revealed evidence of oxidative injury in the liver IR group compared to the other groups.

### 2.2. Liver and Renal Injury Are Associated with Upregulation of Serum NGAL

In this murine model, plasma levels of NGAL at termination were normal in the control group shams and animals receiving RIPC alone. Liver ischaemia reperfusion was associated with increased plasma NGAL at two hours post reperfusion (Figure 1d). This early increase in plasma NGAL shows temporal correlation to the development of liver and renal injury in the model.

### 2.3. With Liver IR Injury and AKI, NGAL mRNA Upregulation Is Seen in the Liver but Not the Kidney. The Liver NGAL mRNA Was Reduced by Pre-Treatment with RIPC

**Figure 2:**
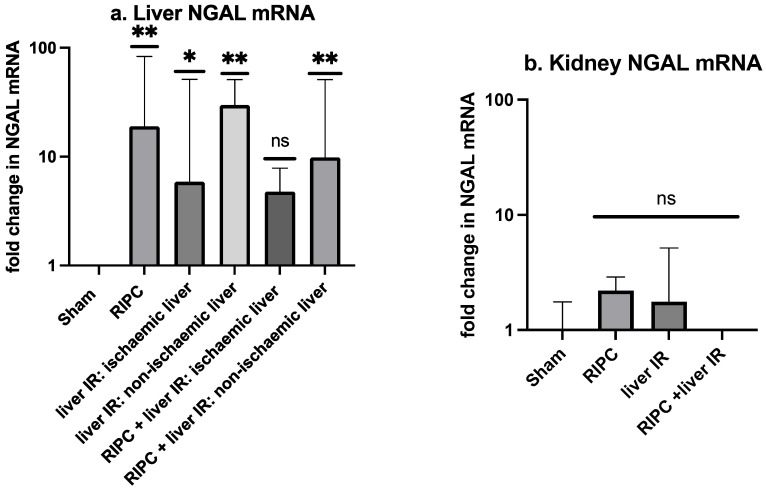
Fold change in NGAL mRNA in each of the experimental groups compared to sham laparotomy for (**a**) liver and (**b**) kidney specimens. Median values and interquartile ranges are shown. Statistical analysis was performed using Kruskal–Wallis and Mann–Whitney tests as appropriate. “Ischaemic” and “non-ischaemic” denotes which liver lobe from the animal was sampled. With regards to statistical significance of result compared to sham laparotomy, “ns” denotes not significant (*p* ≥ 0.05), * denotes *p* ≤ 0.05, and ** denotes *p* ≤ 0.01. Statistically significant *p* values are provided in the text. *N* = 6 mice/experimental group.

Compared to the sham laparotomy, NGAL mRNA was upregulated in the liver tissue following IR. This was evident in liver samples from both the liver lobe subjected to ischaemia (*p* = 0.02) and the non-ischaemic liver lobe (*p* = 0.008) (Figure 2a). Significant upregulation of NGAL mRNA compared to the sham was also seen in the liver samples taken following RIPC alone (*p* = 0.009). Following RIPC + liver IR, significant NGAL mRNA upregulation was only observed in the non-ischaemic liver lobe (*p* = 0.009), with no NGAL mRNA upregulation in the lobe subjected to ischaemia (*p* = 0.13) when compared to the sham laparotomy. RIPC prior to IR may have reduced the liver parenchyma NGAL mRNA in both the ischaemic and non-ischaemic liver, but the reduction was not significant.

NGAL mRNA was not upregulated in the kidney following liver IR, RIPC alone, or RIPC + liver IR when compared to the sham laparotomy group (Figure 2b).

### 2.4. NGAL Expression by IHC in the Liver and Kidney Compared with Plasma NGAL Concentration

**Figure 3:**
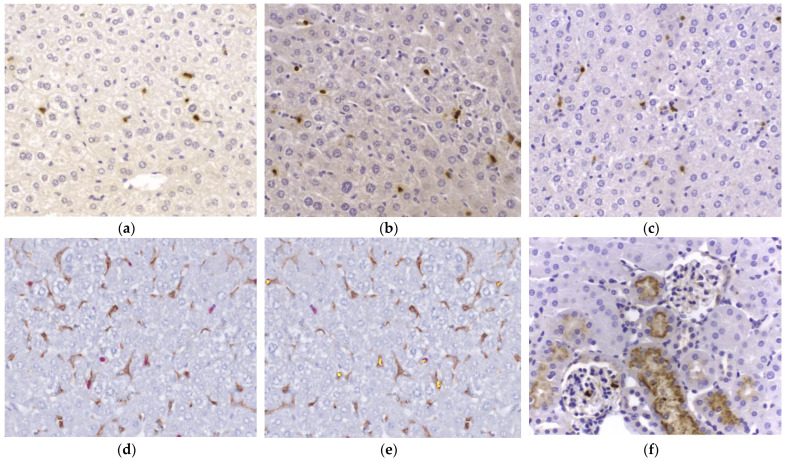
Neutrophil Gelatinase Associated Lipocalin (NGAL) staining on background of haemoxylin/eosin. Cells stained for NGAL are brown, as shown in (**a**–**c**). (**a**) is from a ‘sham’ liver section, (**b**) is from ‘liver Ischaemia Reperfusion (IR)—ischaemic lobe’ and (**c**) is from ‘liver IR—non-ischaemic lobe’. All samples demonstrated NGAL staining with no difference between the groups. Hepatocytes did not stain positively for NGAL and so further staining was performed to identify the NGAL positive cells using F4/80, a marker of macrophages. (**d**) demonstrates co-localization of NGAL (pink) and F4/80 (brown) in a ‘liver IR’ liver section, with co-localization shown by yellow overlay in (**e**). (**f**) NGAL staining within the kidney from the “liver IR” group, with NGAL (brown) positively staining within the apical third of tubular cells. Histological evidence of either liver or renal injury was not demonstrated with H&E staining, and inflammatory cells were not observed. 40× magnification for all images. *N* = 6 mice/experimental group.

To correlate tissue NGAL mRNA expression with serum NGAL levels, histological assessment of six formalin fixed, paraffin preserved (FFPE) liver and kidney specimens from each experimental group were analysed. Liver specimens were selected from the ischaemic and non-ischaemic liver lobes of the same animals to represent an internal control (Figure 3). Firstly, histological assessment of H&E specimens was performed to ensure that the IR protocol had not resulted in liver necrosis. Histology showed no apparent morphological differences between the experimental groups for either livers or kidneys.

NGAL staining was identified within all the specimens. Within the liver, non-parenchymal cells stained positively for NGAL. There was no difference in the number of cells stained for NGAL in any of the experimental groups compared to the sham laparotomy and specifically no increase in the number of cells stained positively for NGAL in the liver IR group (Appendix B). There was also no difference in staining between the ischaemic and non-ischaemic liver lobe samples. In the kidney specimens, NGAL staining centred around renal tubules in all groups. There was no difference in the proportion of renal tubules stained positively for NGAL between the different experimental groups (Appendix B).

### 2.5. In the Liver, NGAL Demonstrates Partial Co-Localisation with F4/80

Hepatocytes did not express NGAL using IHC in any of the groups. NGAL was routinely expressed by another cell population which histologically had the appearance of Kupffer cells. We therefore performed multiplex co-staining for NGAL and F4/80, a marker of macrophages, in the sham laparotomy and liver IR groups. This demonstrated partial, but not complete, co-localisation, suggesting involvement of Kupffer cells in the production of NGAL in this context (Figure 3d,e). Increased co-localisation between NGAL and F4/80 was not seen in any of the experimental groups compared to the sham.

## 3. Discussion

We used an established murine model of partial liver ischaemia reperfusion and in keeping with other studies, demonstrated liver injury with elevations in ALT and AST following the IR insult. Liver injury was reduced by remote ischaemic preconditioning, in the form of repeated 5 min intervals of hindleg ischemia. Again, this has previously been demonstrated in other similar models [40]. In our model, liver IR and liver injury were associated with the development of AKI. The link between liver IR injury and AKI in murine models has been well documented [41], but for the first time, we link the three processes (liver IR injury, RIPC and AKI) and show that RIPC reduces AKI as well as liver IR injury. From an experimental perspective, the ability to attenuate injury by RIPC also increases the utility of this model for the investigation of the mechanisms underlying AKI development following liver IR injury.

Clinically the importance of secondary organ injury is well known. AKI following liver IR injury during liver transplantation is associated with worse overall survival and donor graft function [42]. Clinical data suggests that secondary renal injury occurs within 2 h of liver IR injury [37]. Investigation of the mechanisms of renal injury and new treatments need to focus on the very early changes following liver IR; thus, we chose to study outcomes following two hours of liver reperfusion. Histological cellular changes associated with liver IR injury were not seen in either the liver or the kidney with standard H&E staining at this early time point. Cellular changes take time to develop and were not expected to be demonstrated histologically at this stage. Regarding the biochemical markers of liver IR injury, AST/ALT are widely used markers of liver injury in both the experimental and clinical settings. Creatinine and NGAL provided biochemical evidence of renal injury, which was confirmed by staining for oxidative damage. We did not broaden the model to include multiple longer periods of reperfusion before animal sacrifice because we felt that this data would be less informative and to provide reproducible data would require a greatly increased number of experimental animals. In addition, evidence of cellular changes consistent with renal injury following liver IR at longer time points post reperfusion is already within the published literature [41].

Once the model had been shown to reproducibly produce AKI following liver IR injury, we investigated NGAL in the early stage of liver-IR-induced AKI. Circulating NGAL was increased at 2 h post liver IR injury. This response was attenuated by remote ischaemic preconditioning, in line with the observed attenuation of liver IR injury and AKI, indicating a specificity of the response. To our knowledge, this early time point has only been previously studied in a rat model of liver transplantation, where AST, ALT, NGAL, and creatinine were all elevated at 30 min and 2 h post reperfusion [43]. In mice, the earliest time point studied is 4 h post reperfusion [44], with demonstrated an increase in circulating NGAL at that time point.

We went on to investigate the origin of the serum NGAL increase in this early phase following liver IR injury. We performed immunohistochemistry and evaluated mRNA upregulation within FFPE-preserved livers and kidneys harvested from our murine model. Despite a statistically significant increase in plasma NGAL following liver IR, we did not demonstrate any increase in the number of cells stained for NGAL in either the liver or kidney compared to the sham, RIPC, or RIPC + IR injury. This novel finding raises the possibility that NGAL is not synthesised by either the liver or the kidney in liver IR injury. Published data provides mixed evidence, with some groups reporting a renal origin [45] and others reporting a liver origin [46] for NGAL.

Even more interestingly, hepatocytes did not express NGAL in any of the experimental groups, in contrast to other published studies which demonstrated hepatocyte staining for NGAL in response to liver injury [47]. Instead, the current study findings would be compatible with a study evaluating liver production of NGAL in response to bacterial infection which identified NGAL mRNA upregulation in hepatocytes in combination with increased circulating NGAL but without significant hepatocyte staining for NGAL following inoculation with E coli. The variability in published data related to the origin of NGAL following liver IR injury may be a result of a rapid NGAL release mechanism, as suggested by a hepatic NGAL knockout model [48].

We demonstrate partial co-localisation of NGAL with F4/80 in liver specimens from all four experimental groups, suggesting that Kupffer cells may act as a source for NGAL in murine livers. This is in keeping with previous results [47,49]. The release of NGAL from such a reservoir may explain why an increase in the number of cells stained for NGAL was not observed in our data. The population of F4/80_NEG_, NGAL_POS_ cells in murine livers may resemble monocytes or even neutrophils which have migrated following IR injury. Further work is ongoing to identify the cellular source of NGAL within the murine liver following IR injury.

Despite no change in the number of cells stained positively for NGAL across the experimental groups, mRNA upregulation was observed within the livers but not the kidneys across all the liver IR experimental groups compared to the sham. Some attenuation of NGAL upregulation in response to liver IR was seen with RIPC, although this did not achieve statistical significance. Larger experimental groups may be required to confirm this finding. 

NGAL has previously been reported to be an acute phase reactant [48] released by the murine liver in response to inflammatory or infective stimuli, including LPS [50,51]. It may be that the inflammatory stimulus provided by either RIPC or liver IR induces mRNA upregulation of NGAL. However, this does not explain the relative downregulation of NGAL mRNA following RIPC and liver IR compared to either individual stimulus, and it does not correlate to the serum concentrations of NGAL in each of the experimental groups. This would suggest that NGAL modulation in the model is not simply a reflection of an acute inflammatory response to the experimental intervention. Additionally, within the ischaemic liver lobes, an apparent downregulation of NGAL mRNA is observed from liver IR to RIPC + liver IR, which is in keeping with the reduction in circulating NGAL between these two experimental groups. There may be two mechanisms at work here, a broad “acute phase response” upregulation of NGAL mRNA and a more specific “ischaemia induced” upregulation of liver NGAL mRNA. As only the latter correlates to circulating NGAL, additional control steps may be involved in translation and NGAL release. 

The model of RIPC and liver IR reported here provides some interesting findings with relation to the association between liver IR injury, AKI, and NGAL but also raises further questions regarding the nature of the relationship between the three and specifically the role of the ischaemic versus non-ischaemic liver lobe in the local and systemic response to IR injury and NGAL release. Further experiments in a mouse model may not provide additional insight, as this would require blood and tissue sampling from multiple sites, which would be technically challenging and unlikely tolerated by the anaesthetized rodents. Murine NGAL shares limited homology with human NGAL [52], and whilst NGAL release by the liver as part of a global inflammatory response has been observed in mice, this had not been demonstrated in the human setting. Our ongoing work seeks to address the clinical relationship between liver IR injury, AKI, and NGAL observed in liver transplantation in more detail.

## 4. Materials and Methods

### 4.1. Animals

Mice aged 8–10 weeks C57BL/6 (Charles River, Harlow, UK) were allowed to acclimatise under standard laboratory conditions, with free access to water and chow pellets. This study was performed in accordance with the Animals (Scientific Procedures) Act 1986 and under a project license from the Home Office.

### 4.2. Operative Procedure

**Figure 4:**
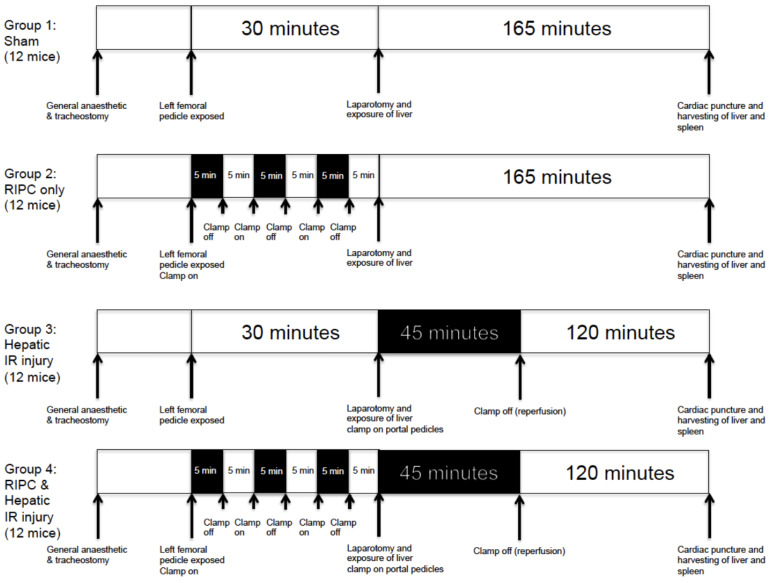
Diagram to show experimental protocol. Mice were randomly assigned to one of four groups. Overall duration of anaesthesia prior to termination was identical across the groups at 240 min.

The experimental procedure is detailed in Figure 4. Mice were subjected to general anaesthetic with 0.4 mL intra-peritoneal Ketamine, Xylazine, and tracheostomy. Further intra-peritoneal doses of Ketamine were given if required. All operative procedures were performed under anaesthesia using a Zeiss (Cambridge, UK), OPMI, 6MD operating microscope. All animals underwent a vertical incision extending from the abdominal midline to the right knee, with exposure of the portal triad and right femoral pedicle.

### 4.3. Sham Procedures/Controls

The femoral vessels and portal triad were exposed without cross clamping. Animals remained under anaesthetic for 195 min (to include the 30 min required for RIPC and 165 min for liver IR required by the other experimental groups) until the end of the experimental procedure when they were terminated by cardiac puncture.

### 4.4. Remote Ischaemic-Preconditioning (RIPC)

In animals assigned to the RIPC group, the femoral pedicle was cross clamped with an atraumatic microvascular clamp for three periods of five minutes duration, each followed by a five-minute interval of reperfusion. The portal pedicle remained unclamped throughout, but 165 min (required for portal clamping and reperfusion in the liver IR groups) of general anaesthesia was administered before termination.

### 4.5. Liver Ischaemia Reperfusion (IR)

Following midline laparotomy, the portal triad and right femoral pedicle were exposed. Anaesthesia was administered for 30 min (required for intermittent hindleg ischaemia in the RIPC groups). Next, an atraumatic vascular clamp was placed across the left and middle portal pedicles, leaving the right portal branch in circulation. After 45 min, the clamp was removed and the liver was re-perfused for 2 h prior to termination, during which the animal remained anaesthetised.

### 4.6. Remote Ischaemic Pre-Conditioning + Liver Ischaemia Reperfusion (RIPC + Liver IR)

Following exposure of the portal triad and femoral pedicle, the femoral pedicle was intermittently clamped, as per the methodology for the RIPC group. Following completion of this phase, the left and middle liver pedicles were clamped for 45 min, as per the methodology for the liver IR group. After 120 min of reperfusion, animals were terminated by cardiac puncture.

Following completion of the experimental protocol in each group, animals were sacrificed by cardiac puncture and exsanguination. Plasma samples were collected and stored at −80 °C until analysis. The liver and left kidney were harvested immediately after termination, fixed in formalin, and embedded in paraffin after 24 h. FFPE samples were then stored at room temperature.

### 4.7. Quantification of Liver and Kidney Injury

Plasma transaminase levels at termination were used as a surrogate marker of liver injury, whilst kidney injury was determined by measurement of plasma creatinine (Jaffe reaction, COBAS Integra 400 plus biochemistry analyser, ROCHE, Welwyn Garden City, UK).

### 4.8. Plasma NGAL

Plasma NGAL levels were measured using the Biolegend Legend Max™ Mouse NGAL ELISA assay (Biolegend, London, UK), as per manufacturer’s instructions.

### 4.9. NGAL Immunohistochemistry of Liver and Kidney Specimens

H&E staining was performed on all samples according to standard protocols. Immunohistochemistry was performed using our local laboratory protocol. FFPE sections were deparaffinised and rehydrated with serial washes in xylene and alcohol through to distilled water. Antigen retrieval was performed by microwaving slides at 640 W for 20 min in 1 L pH 9.0 TRIS ETDA buffer. After washing in Tris Buffered Saline (TBS) with 0.04% Tween-20, endogenous peroxidases were blocked using peroxidase blocking solution (Bloxall^®^, Vector Laboratories, Kirtlington, UK) for 5 min, then rewashed in TBS with protein block (MP-7401, Vector Laboratories). Sections were incubated in NGAL/lipocalin 2 primary antibody (ab216462, Abcam, Cambridge, UK) at a concentration of 1:500 TBS for 1 h at room temperature. After re-washing in TBS Tween, sections were incubated for 25 min in Impress anti-mouse polymer secondary antibody (MP-7401, Vector Laboratories), then washed again in TBS Tween and developed with 3,3′di-amino-benzadine (SK-4105, Vector Laboratories). Sections were then dehydrated, cleared in xylene, and mounted.

### 4.10. Multiplex F4/80—NGAL Immunohistochemistry

To determine co-localisation of F4/80 and NGAL in liver specimens, a multiplex method was used. FFPE sections were deparaffinised and rehydrated with serial washes in xylene and alcohol through to distilled water. Sections were incubated for 30 min at 37 °C in 0.5% Trypsin (MP Biomedical, Santa Ana, CA, USA)/0.5% Chymotrypsin (Sigma, Gillingham, UK)/1% Calcium Chloride (BDH, Lutterworth, UK) in TBS pH 7.6. Sections were bathed in TBS with 0.04% Tween-20, and endogenous peroxidase activity was blocked with peroxidase blocking solution (Bloxall^®^, Vector Laboratories), followed by repeat washing in TBS and blocking with serum block for 10 min (Impress Goat anti-rat detection kit MP7404-50, Vector Laboratories). Sections were incubated for 1 h at room temperature in the F4/80 primary antibody (MCR487R, Biorad, Kidlington, UK) at a concentration of 1:50 TBS, then washed in TBS Tween and developed with 3,3′di-amino-benzadine (SK-4105—Vector Laboratories). After re-washing in TBS Tween, sections were placed in 1 L pH 9.0 Tris EDTA buffer and microwaved at 640 W for 20 min. Sections were soaked in TBS with 0.04% Tween-20, blocked with serum block (MP-5401, Vector Laboratories), and incubated for 1 h at room temperature in NGAL/lipocalin 2 secondary antibody (ab216462, Abcam) at a concentration of 1:500 TBS. After washing in TBS Tween, slides were incubated for 25 min in Impress anti-rabbit AP polymer (MP5401, Vector Laboratories), then rewashed in TBS and developed with fast red substrate (ab64254, Abcam). Slides were rewashed in TBS Tween, rinsed in distilled water, airdried, cleared in Xylene, and mounted.

### 4.11. Interpretation of IHC

NGAL IHC Slides were examined by experienced liver and renal histopathologists who were blinded to the treatment groups. For liver specimens, which demonstrated focal staining, cells stained for NGAL were counted (10× high powered fields). For renal specimens, the intensity of staining and proportion of tubules stained positively for NGAL were determined.

### 4.12. Interpretation of Multiplex F4/80-NGAL IHC

The F4/80 NGAL double epitope IHC were viewed using a Mantra 2 multispectral imaging digital camera and analysed with the inForm^®^ 2.7.0 software (Akoya, Marlborough, MA, USA).

### 4.13. DNA/RNA Oxidative Damage Immunohistochemistry

The sections were boiled for 3 min in citrate buffer (10 mmol/L, pH 6.0) at 95 °C to unmask epitopes, followed by blocking with 1% (*w*/*v*) bovine serum albumin (in PBS with 0.01% (*v*/*v*) Tween 20) for 30 min. Primary and secondary antibodies were diluted in PBS containing 0.02% (*v*/*v*) Triton X-100 and 0.02% (*v*/*v*) Tween 20. Between every protocol step, slides were washed in PBS 0.02% (*v*/*v*) and Tween 20 three times for 5 min. After application of the primary antibody DNA/RNA Damage Ab (15A3) (NB110-96878SS) (1:1000) overnight at 4 °C, sections were incubated for 1 h with the secondary antibody (1:500, AlexaFluor^®^633 goat anti mouse, Invitrogen, Paisley, UK) at room temperature. Finally, the slides were mounted with fluorescent-free mounting medium (Prolong Gold antifade reagent, P36930, Life Technologies, UK). Maximal intensity projections were obtained with an inverted Zeiss LSM confocal microscope using Axiovision 4.8.2 SP 3 software with same settings for each slide.

### 4.14. NGAL qPCR

Total RNA was extracted from formalin fixed, paraffin embedded kidney, and liver specimens, using the QIAGEN RNeasy FFPE kit (cat. 73504, Qiagen, Manchester, UK) according to manufacturer’s instructions. RNA was immediately converted to cDNA using Applied Biosystems (Warrington, UK) ^TM^ High-Capacity cDNA Reverse Transcription Kit (cat. 4368814) and in a G-storm Thermocycler, as per manufacturer’s instructions.

cDNA was stored at −20 °C pending PCR. qPCR was performed using Applied Biosystems™ Power Up SYBR Green master mix and on a Roche Lightcycler^®^ 96 at cycle settings recommended in the Applied Biosystems™ protocol but with 45 cycles rather than the recommended 40 cycles. Negative controls were run on each plate. NGAL primer sequences were obtained from the Harvard Primer bank (https://pga.mgh.harvard.edu/primerbank/, accessed on 21 February 2024). Four primer pairs were tested (Appendix C, Table A1), with selection of the most efficient primer pair. Four normalisation genes were selected from the published literature and http://www.housekeeping.unicamp.br (accessed on 21 February 2024), an online repository of reliable normalisation genes catalogued by tissue and animal (Appendix C, Table A2). Primer sets were tested for efficiency. The two most efficient gene primer sets (P4Hb and Cali) were used for qPCR. Upon analysis of the results, Cali was found to demonstrate greater variability and lower Ct values (sometimes Ct values > 40); thus, P4hb normalised NGAL qPCR values were used for analysis.

## Figures and Tables

**Figure A1:**
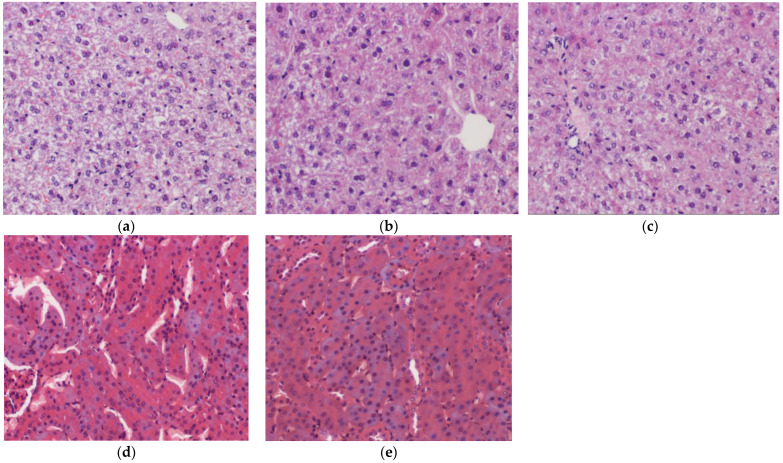
Liver H&E staining from (**a**) sham laparotomy, (**b**) liver Ischaemia Reperfusion (IR)—ischemic lobe and (**c**) liver IR—non ischemic lobe. Kidney H&E staining from (**d**) sham laparotomy and (**e**) liver IR. Histological evidence of injury was not demonstrated in either liver or kidney specimens. All images at 40× magnification.

**Figure A2:**
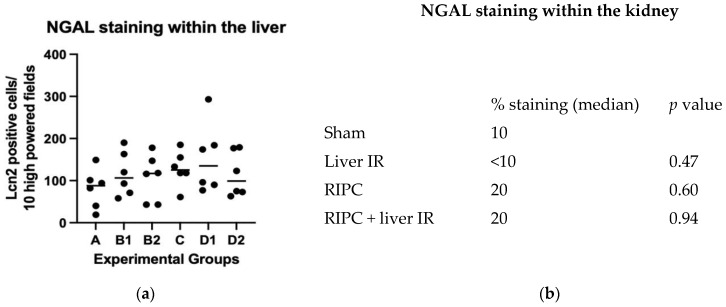
(**a**) Number of Neutrophil Gelatinase Associated Lipocalin (NGAL) staining cells/10 high-powered fields from A: sham laparotomy, B1: liver Ischaemia Reperfusion (IR)—ischemic lobe, B2: liver IR—non ischaemic lobe, C: Remote Ischaemic Pre-Conditioning (RIPC), D1: RIPC + liver IR—ischaemic lobe, and D2: RIPC + liver IR—non ischaemic lobe with no difference between the groups (*p* ≥ 0.05). (**b**) Proportion of tubules stained for NGAL in kidney specimens, with no difference between the groups.

## Data Availability

Data is contained within the article.

## Appendix C

**Table A1:** Primer sequences trialled for Neutrophil Gelatinase Associated Lipocalin (NGAL) qPCR.

Primer Pair	Primer Sequence 5′-3′	Efficiency	Comments
NGAL/1	f-ATGTCACCTCCATCCTGGTCA r-ACAGCTCCTGGTTCTTCCATACAG	80.0%	Dimerization
NGAL/2 *	fGGGAAATATGCACAGGTATCCTC r-CATGGCGAACTGGTTGTAGTC	103.6%	
NGAL/3	f-TGGCCCTGAGTGTCATGTG r-CTCTTGTAGCTCATAGATGGTGC	92.5%	Primer pair does not span intron
NGAL/4	f-GCAGGTGGTACGTTGTGGG r-CTCTTGTAGCTCATAGATGGTGC	108.0%	Primer pair does not span intron

* This primer pair demonstrated the best efficiency without dimerization and was used for qPCR analysis.

**Table A2:** Normalization genes and primer sequences trialled for qPCR analysis.

Normalization Gene	Primer Sequence 5′-3′	Efficiency	Comments
CCT5	f-CTGGGCTCCAAAGTGATTAACA r-TCTCTCCGCTCCATATCTGCC	82.9%	
P4hb *	f-ACCTGCTGGTGGAGTTCTATGC r-ATTGTGGGGTAGCCACGGAC	93.8%	
Cali *	f-TTCTTGGACGGAGATGCCTG r-GGCCCTTATTGCTGAAGGGT	87.9%	
GAPDH	f-GCAATTATTCCCCATGAACG r-GGCCTCACTAAACCATCCAA	38.9%	Primers from literature

* Primers and normalization genes selected for initial qPCR analysis. Cali was found to have Ct values > 40 in multiple samples and so was not used for final analysis.
